# Gender-based adjustment problems of divorcees in Hazara Division, Pakistan

**DOI:** 10.1371/journal.pone.0295068

**Published:** 2023-11-30

**Authors:** Kanwal Rubab, Arif Alam, Ikram Shah, Noor Elahi, Hamayun Khan

**Affiliations:** Department of Development Studies, COMSATS University Islamabad, Abbottabad Campus, Islamabad, Pakistan; University of the Witwatersrand, SOUTH AFRICA

## Abstract

**Introduction:**

Divorce is one of the harshest realities in Eastern societies worldwide because it is an intact component of the elementary social institution of the family. Grievously, divorce rates are escalating with profound ramifications for divorcees in Asia, including Pakistan. Therefore, exploring the challenges after divorce with gender-based dimensions in the Pakistani context was necessary, particularly in Hazara Division.

**Research methodology:**

The study followed a pragmatic approach through snowball sampling and recruited 75 respondents. The data were collected through a semi-structured questionnaire and analysed using IBM SPSS 25. For descriptive statistics, frequencies of quantitative responses were determined using percentages, means, and standard deviations. Then Chi-Square Test of Independence, Principal Component Analysis, and Multivariate Analysis of Covariance were performed to find an association between the dependent and independent variables.

**Results:**

Results demonstrated that most divorcees face children-related issues followed by economic, social, and psychological issues that impede post-divorce adjustment of divorcees. Results showed that the immaturity of divorcees and gender-specific violence specifically for women make it challenging for them to cope with the situation and impede their growth after divorce. Results revealed that more than half of women and a few of men have custody of children after divorce; however, fight over custody of children is another major cause of delayed adjustment. Results presented that gender significantly influences women’s intensity of post-divorce adjustment constraints.

**Conclusion:**

Therefore, it is concluded that regardless of gender, ongoing conflicts with ex-spouses or in-laws made the post-divorce adjustment of divorcees difficult. Divorcees are in a constant tug-of-war between fighting internal dilemmas, pursuing individualistic ideals, and fulfilling societal norms, values, and expectations. This battle complicates and prolongs their adjustments after divorce. The study suggests that institutional, psychosocial, and family support is critical to proactively relieve divorcees from resources and their children.

## Introduction

Dominant cultural norms in a society emphasise the sacredness of marriage and the high value of preserving a family [1]. The family has been a relatively resilient social structure that has survived and adapted over time. It is the central pillar of society as its structure influences society’s social character and personality [2]. Divorce has pervasive weakening effects on the institution of family [3]. However, divorce, a legislatively created and judicially administered process, legally terminates a marital relationship that is no longer considered viable by one or both partners and permits them to remarry [4].

In Pakistan, about 97% of the population is Muslim, which follows Islamic Law or Sharia for marriage. Sharia means ‘the correct path’ in Arabic. In Islam, it is referred to as a divine counsel followed by Muslims to grow closer to God and live moral lives. It is derived from the Quran (considered a direct word of God) and hadith (sayings and practices belonging to Prophet Mohammad (PBUH)). Supplementary sources of Sharia include analogy (Qiyas), the consensus among Muslim scholars (Ijmaa), and independent juristic reasoning (Ijtihad).

Therefore, marriage in Pakistan is a permitted legal union between men and women, a heterosexual union. Culturally, it is a relationship between the husband and wife and collaboration and understanding between their relevant families [5]. However, when marriages undergo troubles, spouses terminate them. It still receives a strong negative response in South East Asian countries, particularly Pakistan, because divorce is taboo and discouraged, especially in rural settings, due to conventional norms and religious reasons [6]. A lack of recognition and respect for divorce erodes family structure. Divorce produces confusion, disintegrates, and introduces chaos in family lives, significantly damaging the social life of conjugal spouses [7]. It deteriorates and affects relationships, leading to futile approaches to managing conflict and weakening the interpersonal skills of stakeholders [8]. Divorce is regarded as an unattractive and detrimental choice. Still, it becomes the ultimate option when the family cannot continue functioning or survive [9]. It is a striking problem in modern society’s social upheavals, killing social harmony at micro to macro levels. It changes social relationships’ nature, structure, and functions by depreciating the social network, affecting a divorcee’s resources and benefits. Divorce transforms a divorcee’s patterns, behaviours, and expectations in social interactions. The altered and damaged social status of divorcees makes them distressed with fear of tomorrow by changing world views on them [10].

Divorce is never a day’s journey but is quite cumbersome [11]. The decline in the well-being of divorcees begins before divorce because it is a process -not an event- and well-being suffers throughout this process [12]. Hence, divorcees manage uncomfortable situations, emotions, and thoughts to merge new experiences in their post-divorce lives. They endure substantial social, economic, cultural, and children-related turmoil, challenging their adjustments [13]. Similarly, it is considered an undesirable act in Pakistan. Not long ago, divorce was considered a social taboo due to societal conservative traits. It was regarded as a social stigma, unacceptable at all costs. It was almost unheard of and interpreted as a shame inflicted on a family’s name a while back. Because of widespread male dominance, women did not want the divorce label attached to them, signifying that the rest of society would consider them social outcasts and family burdens [14].

The culture, tradition, and proper response of the community determine the fate of divorce in different societies. Therefore, all its adverse effects are identified by the societal response to it [15]. Likewise, marriage and divorce are not beyond Pakistan’s societal and institutional influence and contribution. The social identity of being married is a dignified and valued social status, especially a happy and functional marriage, which ultimately becomes a source to enhance an individual’s social and subjective well-being [10].

Additionally, the post-divorce adjustment experience of divorcees is not universal; it varies across different contexts: temporary or life-long experiences [10]. The extent of adjustment issues also varies for divorcees due to differences in their relational, personal, economic, and religious characteristics [16]. These consequences are intricate and extended for a divorcee according to gender, ethnicity, lifecycle, availability of social support, and the existence of children [17]. Thus, divorce affects men and women unequally, depending on a country’s social, cultural, and institutional arrangements [18]. Hence, the responses of each gender to divorce are vastly different, leading to an argument that adjustment is a varied phenomenon for both genders. This is partially true due to gender traits: instrumental men and expressive women [19].

Moreover, every divorcee has a unique experience. Each constructs their narrative describing their interpretations of the events during the marriage, while the divorce happens, and after the divorce [20]. Consequently, divorce as a transitional process changes the relationships, routines, assumptions, and roles of divorcees. Likewise, divorce adjustment is an evolving process rather than a result one has to accomplish [21]. Therefore, divorces are analysed as events; however, they often accompany a lengthy procedure of a relationship termination, including disaffection from the marital partner, conflicts, stress, and even violence [22]. The legal procedures of ending a spousal relationship may last well after both partners perceive the marriage has terminated. Thus, defining and measuring divorce for a divorcee is difficult -when it begins and ceases [23].

Likewise, divorce leaves the divorcees in shock due to altered routines and life patterns. It precipitates emotional reactions like depression, helplessness, and aggression. It leads to socialisation disruptions, making divorcees feel isolated and refused [24]. It brings grief and reduces their physical energy, strength, confidence, and work capacity. They endure embarrassment, hopelessness, restlessness, and feelings of gloom, consequently giving up on everyday life [25]. Divorcees suffer declining health due to residence changes and loss of social support. Pressure to perform the dual role of sole parent and breadwinner promotes unhealthy behaviours and increases mortality risk [26].

Divorced women undergo a psychological crisis of lostness and insecurity caused by decreased material living standards. Insomnia and panic attacks simultaneously affect their health, weakening personality traits over time [27]. However, divorce puts men at a higher risk of health declines due to traditional constructs of masculinity, expecting men to suppress their emotions and not seek help for the strains of divorce. They develop psychological issues of prolonged depression and substance abuse, resulting in impaired well-being and low life expectancy [28]. In contrast, divorced fathers tend to mourn the loss of their ex-wives considerably less than they mourn the loss of their children and their home, family life, and routine [29]. Sadness over the fact that they are no longer there for children and looking out for them fed into feelings of powerlessness and uselessness associated with having their children away from them. Worrying about their children’s well-being leads to losing their appetite [30]. Moreover, infertile divorced women almost wholly lose the remarriage option and face problems throughout their lives [31].

One of the divorce’s most apparent changes is a decline in feelings of competence fuelled by the inability to solve marital problems, make the marriage work, or meet societal expectations of marriage as an institution [29]. Divorced men battle feelings of failure due to a conventional narrative that did not succeed in satisfying the stereotypic male attributes of competence in domestic affairs. They feel disappointed as parents and spouses and express doubts about their ability to adjust well in other relationships [32].

Moreover, the media also portray men as robust, resilient, and confident, not weak or needing help, especially in family matters [33]. Pockets of society still uphold the traditional constructs of masculinity to the detriment of men’s mental health, dictating to them that they should ’support’ and ’protect’ as part of their marital role [34]. Men appear to find it more difficult or unacceptable to articulate their deepest feelings, fears, insecurities, emotional pain, and grief associated with the losses resulting from divorce [35]. Even though society provides conduct models and furnishes roles for married men, it does not do so for divorced men. It makes them unsure how to behave and relate to their children, ex-wives, families, friends, and society [36].

Nevertheless, divorced women seek support from close family members, increasing their psychological well-being [19]. However, in Pakistan, they are perceived as troublemakers and are given only marginal support due to the social stigmatisation of divorce [37]. Ironically, their consanguineous families do not accept them back and cut ties as a punishment for failing to uphold marriage. If taken back, families ill-treat them, considering them a burden [38].

Besides, patriarchal societies marginalise women because of predominant gender inequalities, increasing their vulnerability to poverty [39]. Like in communities where gendered family models are prevalent -the man is the primary breadwinner while the woman works part-time or oversees the housework and childcare- economic burdens of divorce usually fall more heavily on women. Because, particularly in developing countries, marriage is seen as an incentive for women’s financial strength, and divorce takes away her financial stability [40]. Moreover, if women work, they often have lower earnings than men. They are more restricted in labour market opportunities due to fragmented careers after marriage, specifically parenthood [41]. This exerts long-lasting effects on their financial wellness during divorce and later life [42]. Work and family life incompatibility further limits their ability to expand employment activities. Thus, they face acute poverty risks and are more likely to depend on formal or informal support. Besides, ex-spouses’ non-provision of spousal and child maintenance exacerbates their poverty risks [43]. These post-divorce gender inequalities in economic conditions increase mothers’ and children’s vulnerability and decline in their social life. Moreover, divorced mothers navigate a new life while crushing the anxiety over an uncertain future [44]. They experience distress about identity reformulation as single parents [41].

Similarly, divorced men face inevitable consequences due to reformulated identities as single or distanced fathers. They feel disappointed because of diminished immaterial domains of life satisfaction, for instance, emotional well-being and happiness in family life [45]. This is mainly attributable to their reduced opportunities to interact regularly with children [46]. Moreover, while solo-parenting, divorcees are expected to focus on their children’s best interests and leave less room for themselves. They are denied the legitimacy and space to articulate feelings of loss, guilt, and anger [47].

Divorce urges divorcees to restructure the family system and reorient their lifestyle, complicating their adjustment. However, due to financial problems and social taboos, divorced women have no choice but to reside in their parents’ house, tolerating hatred, distrust, aggression, or vengeance [48]. In rural Pakistan, women’s post-divorce rights remain meek as their economic dependence shifts from their husbands to fathers or brothers. On the contrary, for men, problems arise from coping with emotional issues as society expects them to exert masculine traits [49].

Similarly, women endure social challenges like discrimination due to low education and social status [50]. In Iran, they are more vulnerable to the social consequences of divorce [51]. In Pakistan, friends, relatives, colleagues, and neighbours backtrack divorcees. Hence, men struggle to maintain social etiquette, while women feel inferior [52]. Subsequently, women integrate less into their surroundings, influencing their self-esteem. They see everyone from the same prism and lose trust in the opposite gender for remarriage or affection [53].

The rise in divorces subsequently increased the number of women-headed households. These women experience an adverse quality of life, several family and social challenges, especially regarding mental and physical health, delinquency, and increased magnificent social isolation [54]. They suffer from social isolation, limited relationships, mistrust of others due to poverty, their disability to manage a living, and cultural issues of pessimistic views of others [55]. Women’s post-divorce lives often face ambiguities and dangers in Iran, including economic problems, child custody battles, and living independently with despair and depression by facing society’s negative attitude [56]. Rathi and Pachauri (2018) opined that divorce in India results in social rejection for women due to the altered social status of women, they are treated as belonging to a different group, and old friends try to keep their distance due to their social prestige [23].

Ikram and Usman (2022) opined that in the patriarchal culture of Pakistan, divorce is a socially deviant act either by men or women; however, while pursuing divorce, women are more at stake. Divorce brings difficulties for women in self and family formation with new roles and adjustment resources for them, where they are judged by their performance as good wives, mothers, and daughters-in-law [10]. Moreover, Akter and Begum (2012) asserted that women in Bangladesh do not have spatial rights after divorce; they either share their parents’ home, where they feel more emotionally and sometimes physically deprived of themselves and their children. While living alone is a test and trial because of existing patriarchies in private and public life [57].

Moreover, in Pakistan, women are criticised and blamed for divorce regardless of anyone’s fault due to the dominant idea of women’s obligation to make the marriage institution work. Their security lies in marriage [38]. Thus, ramifications such as acute psychological distress and social alienation delay their adjustment process [58]. On top of that, government and society provide negligible support mechanisms complicating their adjustment [59]. The antagonistic court system in Pakistan has made divorce more complicated than it must be. The win-or-lose dichotomic structure of the system puts former spouses, children, and families against each other [5]. Men consider the country’s justice system and social work female-dominated and biased in divorce proceedings, child support, and custody matters. Hence, they feel maltreated and gain negative experiences [29]. Such a condition forces them to have anxiety and sleeplessness [60]. Besides, the post-divorce fatherhood role of a non-custodial financial provider adds pressure on finances and feeds feelings of powerlessness in them upon receiving no recognition [61].

Furthermore, the divorce decision is followed by an extended period of sorrow, grief, and pain. The stressful experience is aggravated since no ritual is prescribed for the loss of divorce experience. Sometimes, divorce-related distress is unacknowledged or unaccepted by others [62]. In Pakistan, divorce results in social isolation of divorcees as readjustment to a new role may prove difficult. They bear quick and loud judgments about their character [63]. Severe socio-psychological outcomes of divorce hit the stakeholders worse, particularly children from divorced spouses. It negatively impacts a child’s mental health and causes behavioural changes affecting social development, as children of today are the human resources of tomorrow [64].

In Pakistan, the prevalent rise in divorce cases has substantially increased the number of divorced and single-parent families. On top of that, society’s challenges make post-divorce adjustment difficult for divorcees. The previously identified problems after divorce are intricate, dynamic, and time-specific and vary from region to region depending on specific socioeconomic, cultural, psychological, religious, and political factors. It is impossible to generalise them in a particular region. Moreover, literature worldwide has ignored gender differences in facing problems after divorce by primarily focusing on women’s perspectives and does not provide separate analyses for each gender. Subsequently, a lack of literature regarding the study subject was identified in Pakistan, specifically in the Hazara Division, making it pertinent to analyse the differentiating views of divorcees on persistent post-divorce adjustment issues. Therefore, this study investigated the post-divorce adaptation challenges faced by divorcees in the study area.

### Theoretical framework

The Divorce-Stress-Adjustment Perspective (DSAP) is commonly adopted to study divorce consequences [65]. Amato (2000) integrated three stress perspectives to offer a model to guide divorce adjustment research by combining their various elements into a DSAP. The DSAP describes divorce as a process that includes the full spectrum of events that precede and follow a divorce. It considers that many stressors are associated with the divorce process, and it suggests that these stressors leave divorcees at increased risk for various problems before and after the divorce [66].

It has a central postulate that divorcees need to adjust to a stressful divorce transition. DSAP views the dissolution process as typically mobilising numerous events that a divorcee experiences as stressful. These stressors, in turn, elevate the risks of adverse behavioural, emotional, and health aftermaths for a divorcee [22]. The severity and duration of these outcomes vary from individual to individual, conditional to the presence of diversified protective and moderating factors. To return to initial levels of well-being and post-divorce adjustment, divorcees need the assistance of certain protective factors such as education, employment, perceptions, and societal attitudes towards divorce. These mediating factors represent how divorce affects divorcees’ functioning and well-being [67].

## Research methodology

The study chose pragmatism as a research paradigm because pragmatist epistemology holds that knowledge is always based on experience [68]. Each person’s knowledge is unique as her/his unique experiences create it. Similarly, every divorcee uniquely experiences divorce depending upon his/her social, economic, psychological, and cultural circumstances [69]. Moreover, the study followed a mixed-method research methodology, simultaneously using quantitative and qualitative approaches to answer research questions while considering the aim and nature of the research due to the sensitive and atypical topic without re-traumatising the participants. This integration provides a deeper understanding of the topic under investigation, thoroughly responds to research questions, and suggests changes to subsequent research designs [70]. This enabled the current study to demonstrate a comprehensive overview of the experiences of divorcees in the study area.

### Study area

The study area of this research is the Hazara Division, Pakistan, comprising eight districts. However, districts Abbottabad, Haripur, and Mansehra are the most prominent concerning area and population. According to the 2017 census, the total population of these districts is estimated to be 3,890,346, which makes up about 64% of the population of the Hazara Division, with a ratio of 50.03% men and 49.97% women [71]. These districts were purposively selected. Moreover, the Haripur district topped the rankings with the best education score of all the districts in the country [72]. According to the Pakistan Social & Living Standards Survey (2019–20), districts Abbottabad, Haripur, and Mansehra were ranked 2^nd^, 3^rd^, and 4^th^ for men’s literacy ratio and 1^st^, 2^nd^, and 3^rd^ for women’s literacy ratio, respectively, in Khyber Pakhtoonkhwa (KP) [73]. Apart from that, the divorce rate in the study area is rising, which is one of the significant reasons behind conducting this research. According to the Pakistan Demographic Survey (2020), there were 31,931 women and 13,278 men divorcees in KP [74]. However, the actual number of divorcees is much higher as people in rural areas do not register their marriages and divorces. Furthermore, these districts are the economic hub of the Hazara Division; they are primarily overcrowded since the people from the peripheries have moved to the main cities either to search for jobs and livelihood or education of children [75].

Moreover, common prevailing causes of divorce are media addiction, relationship traits, the role of families and friends, infidelity, domestic violence, demands and expectations, poverty, and unemployment in these districts [76]. Further, the snowball sampling technique was used to reach out to divorcees as it is pertinent when studying behaviours, perceptions, and descriptions of respondents that cannot be generalised to the entire population. Divorce is a sensitive phenomenon in Pakistan, and people exhibiting social stigma like divorce are considered socially marginalised and atypical, requiring more significant effort to reach out. Therefore, snowball sampling was followed to overcome this issue.

### Sample size and limitations

A total of 75 respondents were reached: 25 men and 50 women. During the data collection process, more than 125 respondents were identified; however, many divorcees did not agree to participate in the survey for different reasons, such as unwillingness to share their experiences and to be reminded of traumatising moments, societal norms, and patriarchy.

Additionally, it was challenging to identify divorcees due to the unavailability of exact official divorce rates and information on divorcees in the Hazara Division, KP, and overall Pakistan.

### Inclusion/exclusion criteria

The primary criterion for including the participants was being divorced, being able to talk, being willing to share personal information, being divorced less than ten years, being with or without children, having an early or late divorce, experiencing any divorce type, and having experienced one or more divorces. Further, divorcees who remarried were also included. Various descriptions regarding the experience being researched are considered necessary to explore the nature of the incident [77]. Therefore, a diverse range of participants was located regarding age and socioeconomic status. Additionally, both genders were chosen to interview due to the possibility that there might be gender-specific differences in responses to divorce.

### Data collection

The triangulation of primary and secondary data collection methods was followed in the study to ensure that the collected data met the needs of the investigation. The primary data from the field was collected through face-to-face interviews using semi-structured questionnaires. The literature published in journals, research reports, articles, and books were read in-depth to discover already known facts about the research problem. Secondary data provided background and foundation for the study.

The principal reason for using the face-to-face interview method in the current research was that the respondents were of diverse social attributes, such as being illiterate, facing difficulty in writing their feelings and perceptions, and being short of time due to increased responsibilities. The face-to-face interviews were conducted through semi-structured interviews, which are halfway between the ends of structured and unstructured interviews [78]. Moreover, the researcher was concerned with finding a way to access their lived experiences as much as possible because divorce is a compassionate and private issue. Subsequently, in-depth interviews were also conducted using semi-structured questionnaires, which helped uncover in-depth and comprehensive information [79].

Informed consent of participants was ensured verbally after explaining the study’s purpose, extent, and limitations and before conducting detailed interviews. After rapport building, the confidentially of their data was ensured by following internationally established ethical standards for research, for instance, by using well-constructed interventions and pseudonyms to keep respondents’ anonymity and pick only relevant information. It was attempted to remain neutral and unbiased while asking sensitive questions. Additionally, having grown up in a similar culture, being aware of norms, gender roles, religious beliefs, and gender-sensitive issues, it was attempted to avoid inconvenience for respondents. Moreover, self-observation through precise and compact interaction was maintained throughout the interview process, keeping in mind the sensitivity of life events of divorce for respondents.

### Data analysis

Initially, data was entered using MS Excel, then analysed using IBM SPSS 25. The descriptive statistics analysis was used to describe the socioeconomic variables of the respondents and summaries their responses. Afterward, the Chi-Square Test of Independence was applied to find significant statistical differences between the responses of divorced men and women regarding their divorce attributes. Finally, Principal Component Analysis (PCA) was performed to investigate the association between the adjustment of divorcees and the constraints they face due to gender differences. The factors that make divorcees’ adjustment difficult were further scrutinised through means and standard deviations.

Finally, a series of Multivariate Analyses of Covariance was performed in this study to find patterns and correlations between several variables simultaneously, such as divorcees’ gender and multiple post-divorce adjustment problems, including social, economic, psychological, and children-related. Respondents’ issues were taken as independent variables, their gender as the dependent variable, and their age as a covariate. Then, a p-value < .05 for one of the outcome variables was considered a significant main effect among the independent groups.

Finally, inductive thematic analysis [80] was employed to analyse the study’s qualitative data. The thematic analysis of post-divorce adjustment challenges of divorcees identified six themes: demographic factors, interpersonal aspects, attitudinal elements, psychological factors, relational ingredients, and supportive resources.

The findings obtained from the interviews were classified into the following themes and subthemes: a) economic problems with subthemes of being a single breadwinner, difficult to get a living cost, economic dependence on family, lack of financial support, and pay/receive child support, b) children-related problems with subthemes of being a divorced parent, economic needs of children, psychological needs of children, growing up children alone, and strained relationship with children, c) psychological problems with subthemes of schizophrenia, feelings of grief/loss, anger, feelings of hopelessness, and lower life satisfaction, and d) social problems with subthemes of societal acceptance of divorce, social stigmatisation, lack of social support, life controlled/behaviour monitored by others, intervention in decisions by others, destruction of personal-familial space, social discrimination, low empowerment, the reaction of people to divorce, treated differently by the community, and discrimination in remarriage.

## Results and discussion

Demographic characteristics of men and women divorcees have been demonstrated in Table 1, showing that the average age of divorcees was 33 years, and the maximum number of divorcees from both groups fell in the 31–40 age group. This may be due to the prevailing opinion that relatively younger individuals divorced at an earlier stage of marriage do not remain divorced for longer. Moreover, the mean age of respondents was 31 at their wedding, and a maximum of men and women got married between 21 and 30 years. However, it is pretty alarming that about 23% of women’s marriages are early.

**Table 1:** Demographic characteristics of divorcees.

Characteristics		Men	Women
Gender		25 (33)	50 (67)
Age *(Mean*: *33 years)*	Up to 20	0	1 (1)
	21–30	5 (7)	19 (25)
	31–40	18 (24)	20 (27)
	41–50	2 (3)	10 (13)
Age at marriage *(Mean*: *31 years)*	Up to 20	0	17 (23)
	21–30	21 (28)	29 (39)
	31–40	4 (5)	4 (5)
Age at divorce *(Mean*: *25 years)*	Up to 20	0	3 (4)
	21–30	9 (12)	23 (31)
	31–40	14 (19)	21 (28)
	41–50	2 (3)	3 (4)
Education level *(Mean*: *9 years)*	Illiterate	3 (4)	8 (11)
	Primary	3 (4)	10 (13)
	Secondary	5 (7)	11 (15)
	Higher Secondary	4 (5)	12 (16)
	Graduation & above	10 (13)	9 (12)
Occupation	Self-employed	3 (4)	11 (15)
	NGO	0	1 (1)
	Daily wages	2 (3)	0
	Government employee	4 (5)	6 (8)
	Business	6 (8)	2 (3)
	Private job	7 (9)	12 (16)
	No occupation	3 (4)	14 (19)
	Student	0	4 (5)
Employment	Before marriage	23(30)	9(12)
	During marriage	21(28)	15(20)
	After divorce	22(29)	38(51)

**Note:** (i) Values on the left are the number of responses, while values in parentheses represent percentages.

At the same time, respondents’ mean age was 25 at their divorce; the majority of men divorced between 31–40 years of age, while a majority of women divorced between 21–30 years of age, implying that early marriages result in early divorces. Furthermore, the respondents’ mean years of education were nine; most men were graduates, while most women were high school graduates. This is because the study area has high literacy ratios in the province.

Likewise, to fully understand the phenomena of divorce and related contexts, the respondents were chosen from diverse professional experiences. Therefore, Table 1 depicts that 15% of women respondents were self-employed. Most of these respondents started working after divorce; most were tailoring and giving tuition to neighbours’ children at home. Moreover, 3% of male divorcees mentioned that they are daily wagers, such as drivers. It was further indicated that 8% of female employees were government employees who served as doctors, nurses, librarians, IT professionals, and school teachers. It was also discovered that 9% of men and 16% of women are in private jobs like teaching in a private school, working in a beauty salon, and other private organisations.

Table 1 further indicates the employment status of divorcees before and during marriage and after divorce. Responses that more than half of women (51%) gain employment after divorce signifying that they mainly suffer more than men in economic realms after divorce. Moreover, the transition of the employment status of more women also supports the view that no formal or informal support mechanism exists in Pakistan for divorced women and their children. Families are either unable or unwilling to support them. Contrarily, men’s employment data shows no considerable variation before and during marriage and after divorce.

### Divorce attributes experienced by divorcees

The divorce attributes experienced by respondents are presented in Table 2. After applying the Chi-Square Test of Independence, a significant statistical difference was inferred between men and women for the variable type of divorce. Among four types of divorces, one of the respondents opted for the option ’wife’s right to divorce.’ This adheres to the generally prevailing practice that different clauses of *Nikahnama* are cut off by religious scholars (*Maulvi*) before the family or bride and groom. It is a cultural practice and a general assumption that the husband does not want to allow his wife to avail the divorce right (clause 18), and the man’s right to divorce is not to be questioned (clause 19). *Nikahnaman* is the marriage contract in Pakistan. It is also the primary component of an Islamic marriage consisting of rights, responsibilities, and favours from the bride and groom and their families. The *Nikahnama* form has four copies duly filled by the *Nikah* Registrar and signed by the bride and groom. One copy of the *Nikahnama* is provided to the Union Council as a public record per the MFLO, 1961.

**Table 2:** Responses of respondents on divorce attributes.

Attributes		Men	Women	χ^2^ value	df	P-value
Type of divorce	Mutual consent	2 (8)	3 (6)	6.84	3	0.130
	*Khula*	9 (36)	14 (28)			
	Triple *Talaq*	14 (56)	32 (64)			
	Wife’s right to divorce	0	1 (2)			
Divorce settled through	Court	10 (40)	17 (34)	8.70	5	2.644
	*Jirga*	1 (4)	1 (2)			
	Religious leaders	1 (4)	2 (4)			
	Elderly	11 (44)	26 (52)			
	Mutual consent	0	1 (2)			
	Still in court	2 (8)	3 (6)			
Receive/pay alimony	Yes	9 (36)	18 (36)	26.35	1	0.000***
	No	7 (28)	18 (36)			
Divorce happened	Accidental	9 (36)	23 (46)	42.08	3	0.001***
	*Wata Sata*	1 (4)	2 (4)			
	Imposed	9 (36)	10 (20)			
	Willingly	6 (24)	15 (30)			
Inclined towards divorce	Yes	9 (36)	9 (18)	7.60	1	4.415
	No	16 (64)	41 (82)			
Divorce history in the family	Yes	4 (16)	12 (24)	38.77	1	0.015**
	No	21 (84)	38 (76)			
Children custody	Yes	3 (12)	28 (56)	55.39	1	0.000***
	No	10 (30)	5 (10)			

**Note:** (i) Values on the left are the number of responses, while values in parentheses represent percentages.

(ii) *df* stands for the degree of freedom.

(iii) *** indicates significant at 1%, ** indicates significant at 5% and * indicates significant at 10%

(iv) *Jirga* is a meeting of a group of elders that has the authority to settle a dispute in a way acceptable to both sides.

(v) *Wata Sata* means exchange marriage system.

Moreover, a statistical difference was found for men and women for the attribute’ divorce settled through’ and ’receive/pay alimony.’ However, most men initiated divorces as ’triple *talaq*’ and settled through the elderly. In contrast, a second significant percentage of divorces was *Khula*, which is the dissolution of marriage initiated by the wife and granted by the court. Besides, the responses of divorcees show that only 36% of men had to pay alimony. The rest of the divorces were ’*Khula*,’ in which wives are restricted from demanding maintenance. Similarly, the responses of divorcees show that only 36% of women had received alimony, while the rest were barred from requiring the same due to *Khula*.

Furthermore, a significant statistical difference was found for men’s and women’s attributes of ’divorce happened.’ According to overall responses, most divorces happen accidentally, which causes post-divorce regret for men. At the same time, men mentioned *Khula* as imposed divorces. Most divorces that happened ’accidentally’ show that lack of patience is rampant in society, leading to drastic consequences. In addition, there was a significant statistical difference between the attributes’ inclined towards divorce’ and ’divorce history in the family’ for respondents indicating that most divorcees were not inclined towards divorce, adhering to the previous result that most divorces happen accidentally. Moreover, from the responses of participants, it is evident that in divorces involving children, primarily mothers had custody.

**Fig 1:**
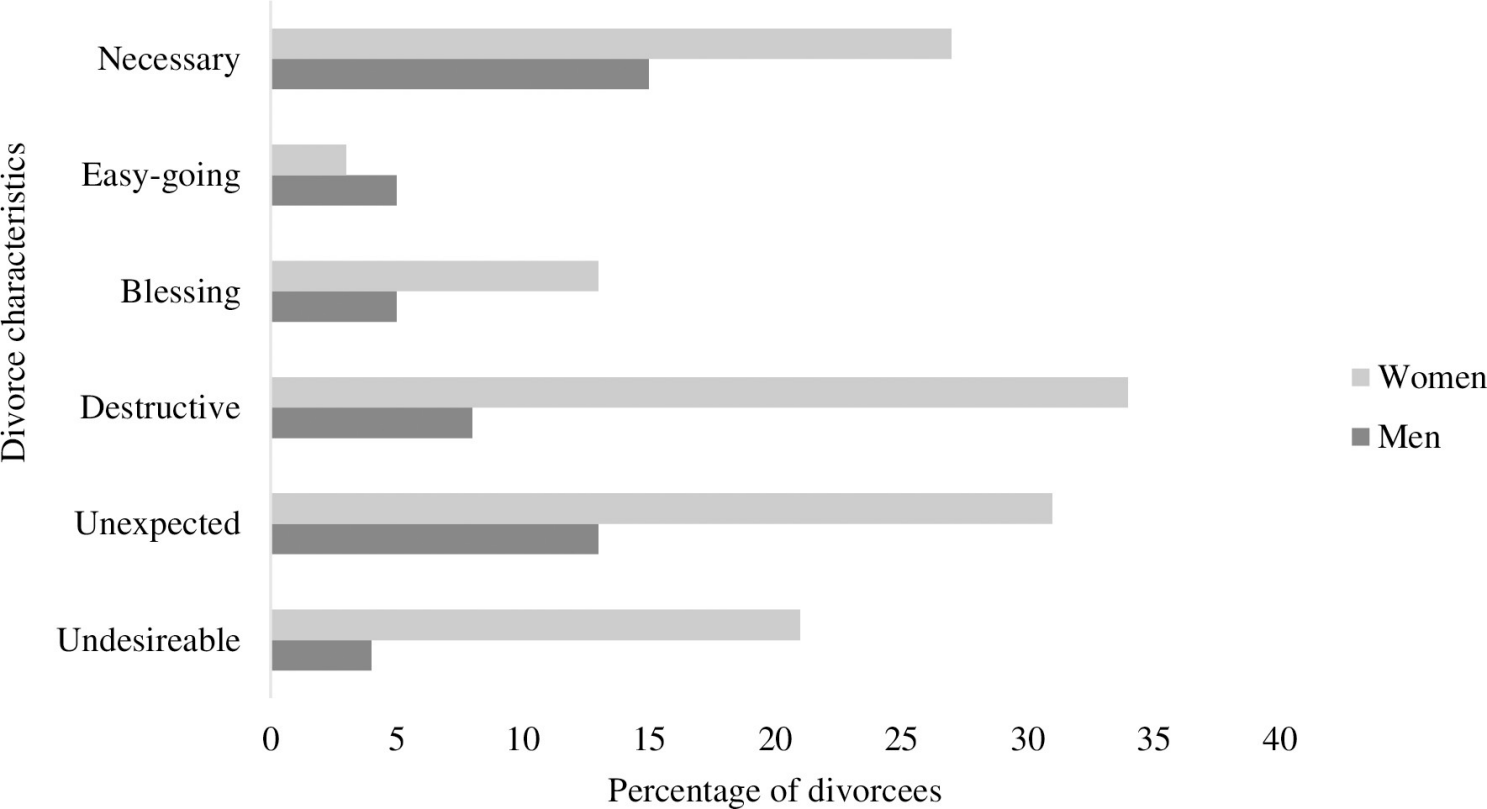
Responses of divorcees based on divorce characteristics.

The distribution of men and women based on divorce characteristics is presented in Fig 1. The figure elaborates that women had more unwanted and displeasing experiences than men. A significant percentage of women expressed their divorce as ’destructive, unexpected, and undesirable,’ while most men chose their divorce characteristics as ’necessary.’ This implied that women, more than men, face problems in post-divorce life in Pakistan.

**Fig 2:**
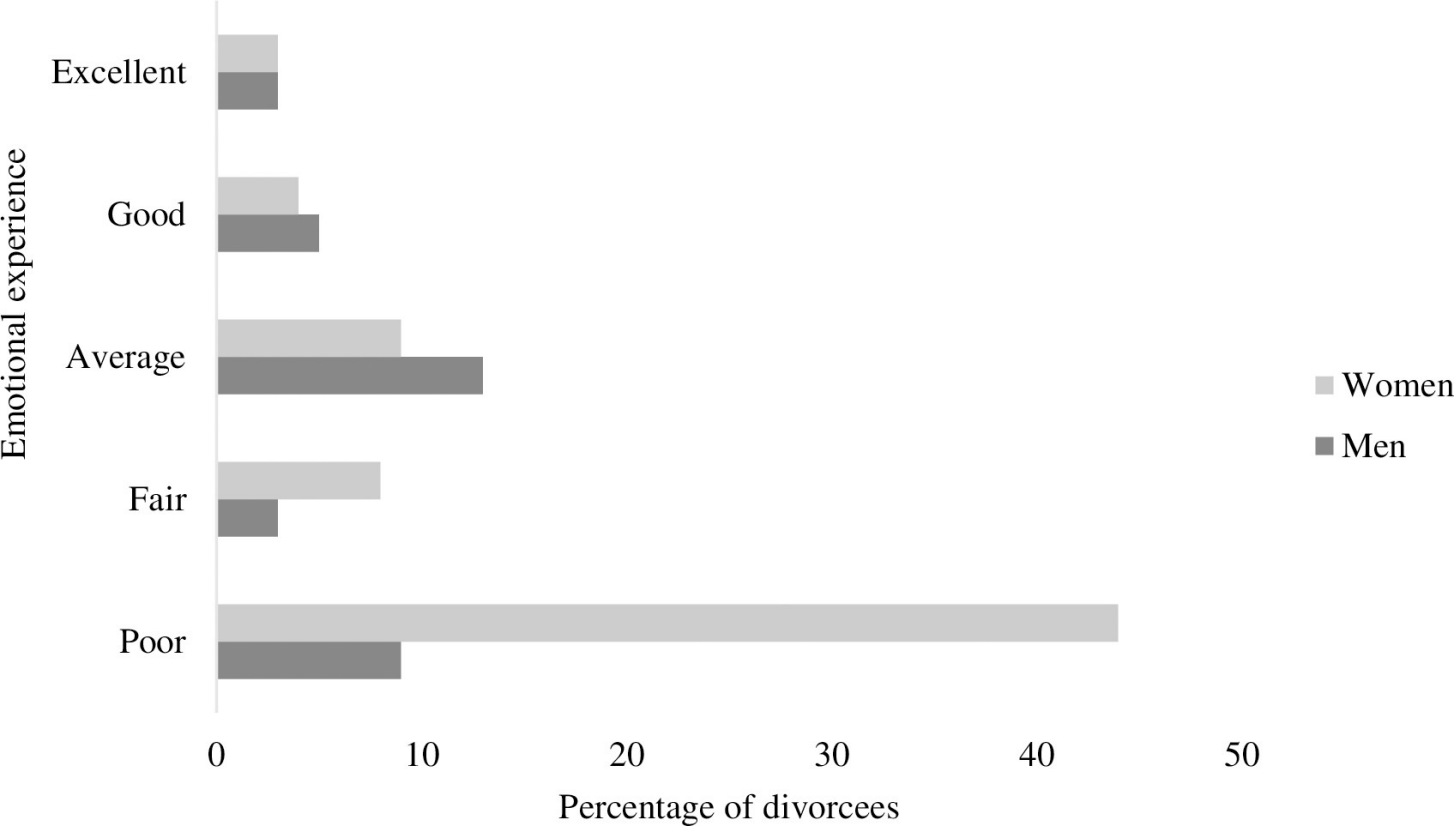
Responses of divorcees based on their emotional experience of being a divorcee.

Moreover, the distribution of men and women based on their emotional experience of being a divorcee is demonstrated in Fig 2. It depicts that a significant percentage of women expressed their emotional experience as ‘poor.’ While most men opted for their emotional experience as ‘average,’ a few respondents selected their emotional experience as ‘excellent.’ This implied that men get more emotional support from families in Pakistan.

**Fig 3:**
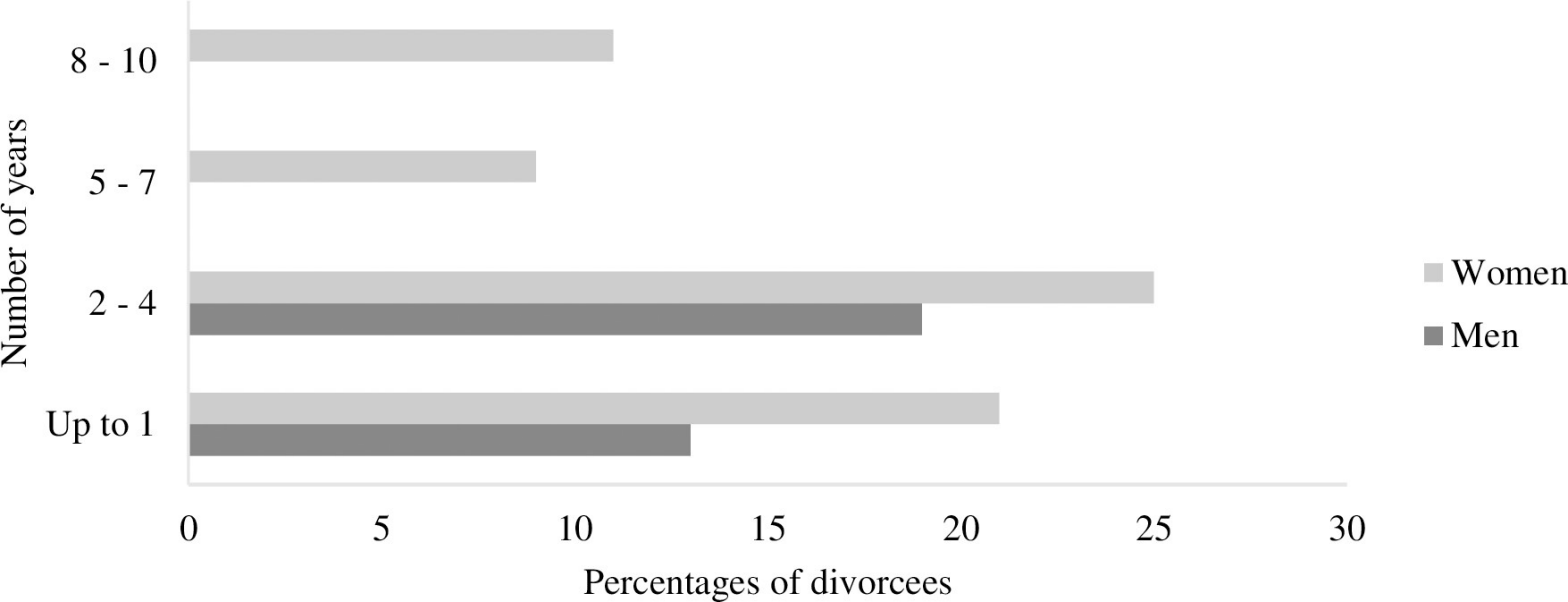
Responses of divorcees based on length of divorce *(Mean*: *3 years)*.

Likewise, Fig 3 presents the respondents’ divorce length in the number of years. According to the responses, the average divorce years is three, and most respondents divorced for 2–4 years. Furthermore, none of the men has more than three years of divorce length, indicating that men do not face obstacles to remarrying, and they opt for remarriage earlier than women in Pakistan.

### Gender-based post-divorce adjustment constraints for divorcees

According to DSAP, divorce does not necessarily bring an end to the stress associated with the end of an unhappy marriage, even for the initiator. Instead, new events and processes emerge at the end of a marriage affecting the divorcee’s emotions, behaviour, and health. Concurrently, this study outlined several post-divorce adjustment constraints that divorcees identified, affecting their later life. The PCA was applied to find the factor structure of post-divorce adjustment constraints, presented in Table 3.

**Table 3:** Principal components factor loadings of post-divorce adjustment problems of divorcees.

	FL	Mean	SD	Rank
**Children-Related Factors**				
Being a divorced parent	0.871	0.43	0.498	12
Economic needs of children	0.838	0.40	0.493	14
Psychological needs of children	0.799	0.28	0.452	22
Growing up children alone	0.765	0.32	0.470	20
Strained relationship with children	0.721	0.05	0.226	26
**Economic Factors**				
Being a single breadwinner	0.870	0.73	0.445	2
Difficult to get a living cost	0.813	0.64	0.483	3
Economic dependence on family	0.787	0.39	0.490	17
Lack of financial support	0.746	0.09	0.293	25
Pay/receive child support	0.732	0.31	0.464	21
**Social Factors**				
Societal acceptance of divorce	0.868	0.57	0.498	7
Social stigmatisation	0.843	0.37	0.487	18
Lack of social support	0.791	0.63	0.487	4
Life controlled/behaviour monitored by others	0.776	0.4	0.493	15
Intervention in decisions by others	0.753	0.6	0.493	6
Destruction of personal-familial space	0.737	0.21	0.412	23
Social discrimination	0.727	0.2	0.403	24
Low empowerment	0.803	0.33	0.475	19
The reaction of people to divorce	0.763	0.45	0.501	11
Treated differently by the community	0.741	0.40	0.493	13
Discrimination in remarriage	0.736	0.73	0.445	1
**Psychological Factors**				
Schizophrenia	0.834	0.40	0.493	16
Feelings of grief/loss	0.826	0.56	0.500	9
Anger	0.797	0.60	0.493	5
Feelings of hopelessness	0.796	0.56	0.500	8
Lower life satisfaction	0.742	0.48	0.503	10

**Note:** (i) FL stands for Factor Loadings, and SD stands for Standard Deviation

(ii) All dependent variables are taken as binary, where 1 equals yes, and 0 equals otherwise

The extracted factors for post-divorce problems and their respective factor loadings were placed according to the highest to lowest. It was revealed from the list of identified principal components that children-related factors have the highest factor loadings, followed by economic, social, and psychological factors. This is because parents prioritise children’s needs more than their own, and responses of divorcees identified that more than half [28] women and three men have the custody of children, and 15 divorcees were distant parents. Moreover, all of the factor loading values are positive, indicating that these are positively correlated with the principal components of the study.

Table 3 indicates the estimation of means and standard deviations of post-divorce adjustment problems. Moreover, Post-divorce problems identified by respondents are further presented in Table 4, and means and standard deviations are calculated for overall results and separately for men and women. The p-values indicate that gender significantly affects post-divorce psychological, social, children-related, and economic aspects for divorcees and complicates their adjustment. Moreover, the MANCOVA results show that greater mean values of factors for women demonstrate that gender significantly influences the intensity of their adjustment problems after divorce more than men.

**Table 4:** Post-divorce adjustment problems of divorcees.

Factors	Men		Women		Overall		P-value	η_p_^2^
	Mean	SD	Mean	SD	Mean	SD		
Economic	1.96	0.73	2.7	1.11	2.45	1.06	0.001	0.138
Children-related	1.8	2.06	3.96	3.02	3.24	2.91	0.000	0.157
Psychological	6.6	1.89	9.3	2.48	8.4	2.63	0.000	0.244
Social	4.4	1.50	8.48	1.97	7.12	2.66	0.000	0.531

**Note:** (i) SD stands for Standard Deviation.

The top-ranked adjustment problem identified by respondents after divorce includes discrimination in remarriage. This is because none of the women respondents could remarry, adhering to the general perception of society that women are troublemakers in marital relationships. Besides, the existence of children impedes the social adjustment of women more than men when women want to remarry. However, the gradual emergence of liberal social values in a conservative society like Pakistan has altered social taboos [81]. As an illustration, a mother of a daughter, working since divorce to send her daughter to school, opined how the presence of her daughter is affecting her post-divorce adjustment,

*’My parents want me to remarry as soon as possible, but not a single marriage proposal has been received since divorce because I have a daughter with me.’*

Moreover, higher reporting of overall outcomes and a higher number of problems by women than men postulate that this could be due to the women being more in tune with their inner world and more readily expressive than men. Furthermore, the views of men demonstrate that the narrative in traditional societies like Pakistan that women bear the highest burden of divorce than men is inaccurate; men undergo persistent adjustment issues after divorce.

Responses of most women revealed that they face post-divorce economic problems more than men. Among them, most women were less educated and did not earn before or during the marriage. Moreover, after divorce, most women had custody of children, so they had to meet their children’s needs. It was identified that less educated or illiterate women earn insufficient money due to less or no experience; therefore, they find it challenging to manage household expenses and children’s education. Consequently, they perceive their post-divorce situation deteriorated more than men because men are free of marital and children’s responsibilities. Similarly, Qamar and Faizan (2021) concluded that most uneducated women do not receive financial support from their families and have no proper source of income [37]. Contrarily, it is evident from the analysis of the current study that men pay more immaterial tolls after a divorce than monetary costs, and women pay immaterial as well as monetary costs.

Apart from economic problems, analysis of responses from divorcees disclosed that psychological problems impede their post-divorce adjustment. For instance, hopelessness inhibits adjustments of recently divorced women because of unfulfilled dreams and hopes of marital life. Moreover, according to the men and women whose divorces involve the court and cases are still pending, they feel angry due to uncertainty in divorce proceedings. The responses further identified that women with custody of children feel depressed because they worry about an uncertain future and feel heartbroken due to dependence on others for basic needs. A less educated custodial mother of three children expressed that,

’*I was severely depressed for years because divorce destroyed my dream of a happy family*. *My world turned upside down*. *I felt I had lost everything I had worked so hard to build and did not know what the future would hold*.*’*

In contrast, all men feel depressed because of losing a life partner, and distanced fathers feel guilty about separation from their children. Besides, almost all men shared that the loss of marriage brought grief and anger due to unfulfilled desires and marital expectations at the beginning of a marriage. At the same time, women self-blame for failing to keep their marriage vows. However, divorce stigmatisation, lack of financial support, being a divorced parent, and blame for divorce were associated with increased symptoms of schizophrenia in divorcees, significantly in younger and less educated women. The views of men further revealed that divorce left them with schizophrenia due to altered routines and life patterns.

Likewise, most participants reported that lower life satisfaction further compounded their adjustment affecting their concentration, and they fell into negative thought patterns. Dependent women felt profound shame that they had brought disrepute to themselves and their families. However, it was found that men did not respond adequately to the questions about widespread problems after divorce, especially psychological ones. It may be due to their instrumental traits and masculine constructs of society; men either did not feel many psychological issues or did not share their actual experiences during interviews. Moldovan (2014) supports the view by saying that an incomplete expression of personal pain delays divorce rehabilitation and causes difficulties in post-divorce relations [82]. A less educated woman opined,

’*After the divorce*, *I had to return to my father’s house with my two children*. *I felt like a parasite there*. *My brother was not comfortable with the increase in household expenses*. *My sister-in-law used to propagate anything my children did as offensive*.*’*

Moreover, the responses of divorcees disclosed that women undergo more social issues such as family members controlling life, outsiders monitoring behaviour, intervention in decisions by others, decreased living standards, and social discrimination. However, the magnitude of these effects varies significantly across countries. These adverse effects tend to be weaker in familistic than in individualistic countries and countries where divorce is more common [83]. Therefore, women in the current study were more exposed to these issues because of the familistic nature of Pakistani society. Since Pakistani society believes in consanguineous marriages, a woman who divorced after a love marriage opined,

’*My family broke all relations with me right after my marriage*. *So*, *after the divorce*, *I could not get their help*.*’*

For instance, women having no or minor children found it challenging to live independently. If they do so, they face all kinds of gossip and scandals and feel insecure socially and physically. This is primarily because customs and culture in Pakistan require a woman to have a male family member in a protective and supervisory role. Women willingly or coercively turn to their parents as a safe place for themselves and their children. Unfortunately, the family does not accept them open-heartedly due to societal and cultural strictness, lack of income to provide food and shelter, and the view that they brought them dishonour. Ultimately, it becomes inevitable for women to move out or bear miseries. It brings reparation in personal-familial space for them to express themselves freely and escape oppression. Few women lived in Women Crises Centers (*Darul Aman*) established by the KP Government in District Abbottabad, Haripur, Mardan, and Peshawar to provide shelter to women affected due to divorce, social injustices, sexual violence, kidnapping, early marriages, and other problems. These centres help in the adjustment of destitute divorced women by providing accessible shelter facilities, food, basic needs, a qualified nurse for in-time health coverage, hiring Advocates voluntarily, religious teachers to give spiritual therapy by Quran, Hadis, and Prays, vocational training likes cutting, stitching, embroideries, knitting, professional psychological counselling, by conducting *Jirgas* for inmates and their families reconciliation depending on the situation, and by providing indoor reconciliation activities. They feel socially excluded and discriminated against, exhibit rude and aggressive behaviour, and lose patience and tolerance. However, men in this study did not have such post-divorce experiences.

Moreover, upholding traditional and dominant family values, divorcees are accountable to families about divorce in Pakistan and deal with successive feedback and responses. Hence, society attaches a considerable stigma to divorce and considers it against societal norms. Remarkably, women respondents, whether educated, employed, or having children, lose social status and respect in tight-knit families. A few men perceived that they also underwent such adversities at a low level. Moreover, it was established from the opinions of women that *Khula* elevates their adjustment problems because they became a source of resentment for their families. Thus, most women consider it a discriminated society where men are powerful and absolved of all blame. for instance, a woman having four daughters living with her parents opined that divorce identity is making post-divorce adjustment difficult for her and her daughters,

’*Being known as a divorcee gives pain to me*. *Everyone notices my daughters and my movements and tries to advise me*. *I feel my daughters and myself very vulnerable*.*’*

Furthermore, women revealed that societal non-acceptance of divorce led to social disruptions for them more than men. Divorcees from both genders shared that they isolated themselves socially due to emotional setbacks and the curiosity of others. The tug-of-war between pursuing individualistic ideals, fighting internal dilemmas, and fulfilling societal expectations, norms, and values complicates and prolongs their adjustments after divorce. They further mentioned that they needed the support of family members, friends, and relatives to curb the negative impacts of divorce and provide comfort and solace. However, significantly less educated women who initiated the divorce, revealed that they did not have the social support that men enjoy, mainly due to the gender-based constructs and violence in society and the generally accepted view of society that women are the main culprits of divorces. Another Pakistan based research supports the view and opines that women face rejection by their natal families because parents do not favour divorce under societal pressures, even if they are cognisant of women’s difficulties in their marriages [38].

Additionally, the analysis of responses from men and women determined that post-divorce children-related factors affect women’s adjustment more than men partially. The research on the role of the judicial system of Pakistan also asserted that Pakistan’s legal system prefers custody of children entrusted to women rather than men [84]. Ironically, although considering divorced women stigmatised, society also expects that children should be in the custody of women because a social role prescribed by the community for mothers is to be the first person to give an account of children’s education and good behaviour. Thus, responses of divorcees in the current analysis revealed that divorce most often redistributes parental responsibilities undertaken by divorced mothers. Thus, tensions about children’s economic and psychological needs and raising children alone increase vulnerability and deteriorate the social lives of divorced mothers and children.

Moreover, divorcees claimed that men left children willingly with their mothers, considering children to be problematic like their mothers. On top of that, most men opted for a second marriage earlier than women despite not having resolved the prior marriage’s dowry and alimony issues. Still, men who had custody of children mentioned encountering enormous problems being divorced parents like women. Furthermore, respondents’ views disclosed that the custodial parent feels overburdened by the presence of children. They believe that fear of improper upbringing keeps them in constant distress. The absence of other parents and changes in the home environment and residence establish strained relationships between single parents and children. Besides, custodial women who are also single breadwinners revealed that it was challenging to manage children’s post-divorce behavioural conduct problems and emotional disorders of loneliness and depression. They remain busy earning outside the home, leading to a decreased focus on children’s schooling. Therefore, they experience twofold adjustment issues: intrapsychic conflicts and their role as single divorced parents.

## Conclusion

This study focused on determining post-divorce adaptation constraints that affected divorcees’ life. Results show that most women get married at an early age, and immaturity causes divorce. In contrast, immaturity and gender-based acts of violence make it challenging for them to cope with the situation and impede their growth after divorce. Results revealed that more than half (56%) of women and only 12% of men have custody of children after divorce. Due to this, custodial parent’s adjustment issues get multiplied. The results of the Principal Component Analysis adhered to this and revealed that children-related factors are the top-ranked adjustment problems, followed by economic, social, and psychological factors. However, most men got remarried and readjusted while women could not despite striving. They face discrimination in remarriage due to the presence of children and society’s general perception that women are troublemakers in marital relationships, causing divorces. Multivariate Analysis of Covariance depicted that gender significantly influences women’s intensity of post-divorce adjustment problems.

Results demonstrated that in 25% of divorces, females approached courts at some stage of divorce regarding child maintenance and custody and dowry issues. Due to this, both men and women experience low levels of adjustment. Even though most respondents have the same extended families with ex-spouses due to consanguineous marriages, many women consulted courts because their ex-spouses initially divorced them but later refused to be safe from paying alimony. This indicates that divorce does not necessarily end marital stress but brings a new set of stressors for both ex-spouses approving the central notion of the Divorce Stress Adjustment Perspective. Therefore it is concluded that regardless of gender, ongoing conflicts with ex-spouses or in-laws made the post-divorce adjustment of divorcees difficult. Divorcees find themselves in a constant tug-of-war between fighting internal dilemmas, pursuing individualistic ideals, and fulfilling societal norms, values, and expectations. This battle complicates and prolongs their adjustments after divorce.

The study recommends that awareness regarding intact families should be imparted to the masses, which will improve the quality of marital relationship quality and help curb the divorce rates and their ill consequences. Moreover, improved educational attainment of women will lead to better employment opportunities, resulting in increased bargaining power and improving their position in the home of their husbands and their natal families. The study suggests that institutional, psychosocial, and family support is critical to proactively relieve divorcees from resources and their children.
